# Inhibition of Stat3 signaling pathway by nifuroxazide improves antitumor immunity and impairs colorectal carcinoma metastasis

**DOI:** 10.1038/cddis.2016.452

**Published:** 2017-01-05

**Authors:** Ting-Hong Ye, Fang-Fang Yang, Yong-Xia Zhu, Ya-Li Li, Qian Lei, Xue-Jiao Song, Yong Xia, Ying Xiong, Li-Dan Zhang, Ning-Yu Wang, Li-Feng Zhao, Hong-Feng Gou, Yong-Mei Xie, Sheng-Yong Yang, Luo-Ting Yu, Li Yang, Yu-Quan Wei

**Affiliations:** 1 Department of Liver Surgery and Division of Digestive Diseases, State Key Laboratory of Biotherapy/Collaborative Innovation Center for Biotherapy, West China Hospital, West China Medical School, Sichuan University, Chengdu, China; 2 Department of Pharmacy, Xinqiao Hospital, Third Military Medical University, Chongqing, China; 3 Department of Abdominal Cancer, Cancer Center, West China Hospital, West China Medical School, Sichuan University, Chengdu, China

## Abstract

Colorectal carcinoma (CRC) is the one of the most common cancers with considerable metastatic potential, explaining the need for new drug candidates that inhibit tumor metastasis. The signal transducers and activators of the transcription 3 (Stat3) signaling pathway has an important role in CRC and has been validated as a promising anticancer target for CRC therapy. In the present study, we report our findings on nifuroxazide, an antidiarrheal agent identified as an inhibitor of Stat3. Our studies showed that nifuroxazide decreased the viability of three CRC cell lines and induced apoptosis of cancer cells in a concentration-dependent manner. Moreover, western blot analysis demonstrated that the occurrence of its apoptosis was correlated with the activation of Bax and cleaved caspase-3, and decreased the expression of Bcl-2. In addition, nifuroxazide markedly impaired CRC cell migration and invasion by downregulating phosphorylated-Stat3^Tyr705^, and also impaired the expression of matrix metalloproteinases (MMP-2 and MMP-9). Furthermore, our studies showed that nifuroxazide also significantly inhibited the tumor metastasis in lung and abdomen metastasis models of colon cancer. Meanwhile, nifuroxazide functionally reduced the proliferation index, induced tumor apoptosis and impaired metastasis. Notably, nifuroxazide reduced the number of myeloid-derived suppressor cells in the blood, spleens and tumors, accompanied by the increased infiltration of CD8^+^ T cells in the tumors. Importantly, a marked decrease in the number of M2-type macrophages in tumor in the abdomen metastasis model was also observed. Taken together, our results indicated that nifuroxazide could effectively inhibit tumor metastasis by mediating Stat3 pathway and it might have a therapeutic potential for the treatment of CRC.

Colorectal carcinoma (CRC) is a malignant neoplasm with a high and increasing incidence and a high mortality.^1^ According to statistics, the colon or the rectum cancer is the second most common cancer that causes cancer deaths among males and females worldwide.^2^ Approximately 134 490 new colon or rectum cancer cases were detected in the United States in 2016 and an estimated 49 190 patients died from this disease in the same year.^3^ In addition, the prognosis of colorectal cancer (CRC) patients is based on the depth of tumor invasion and lymph node metastasis.^2, 4^ Moreover, ~50% of patients with CRC develop metastases, and most of these patients have unresectable tumors.^5^ Although there have been advances in surgical and chemotherapy of CRC, the overall survival percentage has not changed much in recent years and has attracted worldwide attention.^5, 6, 7^ Therefore, there is a need for better treatment approaches for CRC.

It is widely known that there are some specific genetic alterations that are found in a relatively high percentage of CRC, such as tumor suppressor genes and cytokines, including Ras, Src, p27^kip1^, p16^ink4a^, p53 and interleukin.^1, 8, 9^ Moreover, various signaling pathways have been implicated in the development and progression of CRC, involving receptor tyrosine kinases (e.g., epidermal growth factor receptor, vascular endothelial growth factor receptor, fibroblast growth factor receptor, and platelet-derived growth factor receptor) and downstream signaling cascades (RAS-RAF-MEK-ERK and PI3K-PTEN-AKT-mTOR).^5, 10^ Notably, these abnormalities involve the signal transducer and activator of transcription 3 (Stat3) signaling pathway.^11^ In fact, constitutive activation of Stat3 has been detected in many cancers, including breast cancer, lung cancer, melanoma and CRC, but is not required for the function of most normal cells.^12, 13^ Stat3 is a point of convergence for multiple oncogenic signaling pathways. Besides, Stat3 as a proto-oncogene could regulate the fundamental cellular and biological processes.^12^ In response to cytokines or growth factors, activated Stat3, as a nuclear transcription factor, has a critical role in regulating genes involved in proliferation, apoptosis, survival, angiogenesis, invasion and metastasis, as well as genes encoding key cancer-promoting inflammatory mediators.^14, 15, 16^ Meanwhile, Stat3 can be manipulated to augment innate and adaptive immune responsiveness to tumors by mediating the accumulation of myeloid-derived suppressor cells (MDSCs) and many other tumor-associated immune cells.^17, 18^

In case of CRC, existing evidences demonstrate that Stat3 is an important factor related to tumor cell growth, survival, invasion and poor prognosis of human colorectal adenocarcinoma.^1, 4, 6^ Moreover, activation of Stat3 is correlated with the overexpression of cyclin D1 in CRC. In addition, a significant correlation was also shown between Stat3 and both survivin and Bcl-xl expression in CRC.^6^ Furthermore, increasing evidences demonstrated that knocking down Stat3 expression by specific siRNA or small molecules could suppress the growth of CRC cells *in vitro* and *in vivo*.^19, 20^ These data all suggest that Stat3 inhibition provides a rational approach to the treatment of CRC. Although much effort has gone into the development of Stat3 inhibitors and series of Stat3 dimerization inhibitors have been discovered via both computational and experimental methods, so far no Stat3-targeting drug was approved by the Food and Drug Administration.^21, 22^

The development of new safer and more effective drugs is an expensive and time-consuming process, and one solution is to find new uses for existing drugs.^23, 24^ Because of the safety profiles and known pharmacokinetics, the existing drugs could be approved by regulatory agencies for human use much faster and easily.^24^ Nifuroxazide is an oral nitrofuran antibiotic that decreases the viability of multiple cancer cells by inhibiting the phosphorylation of Stat3.^25, 26, 27^ Moreover, nifroxazide as an antidiarrheal agent is used elsewhere, although it is not currently approved for use in the United States.^28^ However, the function of nifuroxazide on CRC and its related molecular mechanism have not yet been investigated. Considering the effects of Stat3 in CRC, we hypothesized that nifuroxazide might be useful in the treatment of patients with CRC.

To verify this hypothesis, we evaluated the biological activities of nifuroxazide in CRC *in vitro* and *in vivo*. Our results implied that nifuroxazide could inhibit the viability of CRC cells by inducing apoptosis via the reactive oxygen species (ROS)-mitochondria apoptotic pathway. We also found that nifuroxazide could impair cell migration and invasion. Importantly, nifuroxazide could repress tumor metastasis in the lung and abdomen metastasis models by reducing immunosuppressive cells and enhancing antitumor immunity. These data suggested that nifuroxazide could be a new agent for the treatment of CRC.

## Results

### Nifuroxazide inhibited CRC cell proliferation and induced CRC cell apoptosis

**Figure 1:**
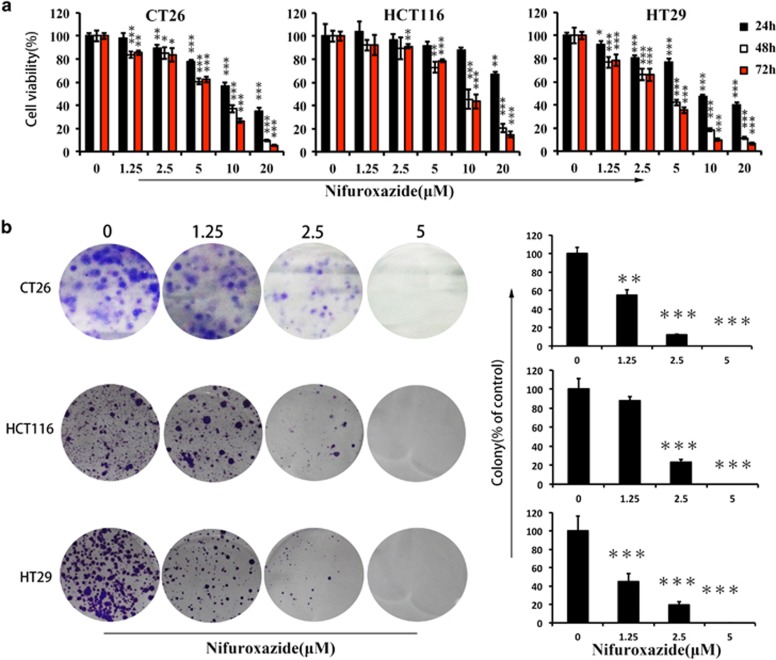
Nifuroxazide reduced viability in CRC cancer cells. (**a**) CRC cell lines CT26, HCT116 and HT29 were treated with different concentrations of nifuroxazide for 24, 48 or 72 h and the cell viability was measured by the MTT assay. Each point represents the mean±S.D. for at least three independent experiments (*P<0.05, **P<0.01, ***P<0.001 *versus* vehicle control). (**b**) The effects of nifuroxazide on colony formation in three CRC cell lines for 12 days; the statistical results of colony formation assays were presented as surviving colonies. Data are expressed as mean±S.D. from three experiments (*P<0.05, **P<0.01 and ***P<0.001)

Constitutive Stat3 activation seems to be one of the critical pathways in CRC and is considered to have an important role in CRC carcinogenesis.^19, 20, 21, 22^ Moreover, Stat3 phosphorylation at tyrosine residue 705 (Y705, p-Stat3) has high expression in human colon cancer cell lines.^11^ To investigate whether nifuroxozide has direct effects on CRC tumor cells, we tested the proliferation inhibition caused by nifuroxazide in HCT116 and HT29 human CRC cell lines, as well as murine CT26 tumor cells by the MTT (3-(4,5)-dimethylthiahiazo(-z-y1)-3,5-di-phenytetrazoliumromide) assay. Treatment of HCT116, HT29 and CT26 cells with various concentration of nifuroxazide for 24, 48 and 72 h, respectively, resulted in a decrease in the cell viability (Figure 1a). These results suggested that nifuroxazide could inhibit CRC cell viability in a concentration- and time-dependent manner. To further confirm whether nifuroxazide could inhibit viability of CRC, clonogenic assay was performed to visually assess the antiviability activity of nifuroxazide. As shown in Figure 1b, the clonogenic assay clearly showed that clone formation of HCT116, HT29 and CT26 cells was reduced in a concentration-dependent manner after exposure to nifuroxazide. In addition, the size of the colonies treated with nifuroxazide was significantly smaller than the negative control group. Taken together, these results suggested that nifuroxazide had a strong cytostatic and cytotoxic effects on CRC cells.

To examine whether the antiviability activity of nifuroxazide on CRC cells was associated with apoptosis. Hoechst 33358 staining assay showed that nifuroxazide altered the morphology of HCT116, HT29 and CT26 cells and induced apoptosis after 24 h treatment, with the features of a bright blue fluorescent-condensed nuclei and nuclear fragmentation. Besides, these changes were concentration-dependent (Supplementary Figure 1).

**Figure 2:**
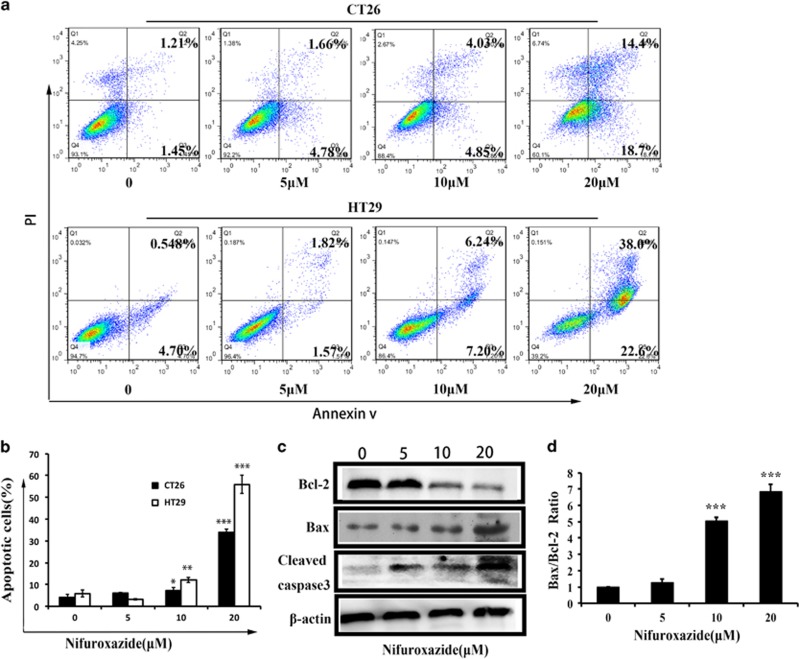
Nifuroxazide-induced apoptosis of CT26 and HT29 cells. (**a**) CT26 and HT29 cells were treated with indicated concentrations of nifuroxazide for 24 h, respectively, and then were analyzed by FCM using the Annexin V/PI dual-labeling technique. Each point represents the mean±S.D. for at least three independent experiments (*P<0.05, **P<0.01, ***P<0.001 *versus* vehicle control). (**b**) Statistical results of apoptosis assays, including the early apoptotic cells and the late apoptotic cells. Data are expressed as mean±S.D. from three independent experiments (*P<0.05, **P<0.01 and ***P<0.001). (**c**) Western blot analyses of CT26 cells treated (24 h) with different concentrations of nifuroxazide; the expressions of cleaved caspase-3, Bcl-2 and Bax were determined by western blot with *β*-actin was used as a standard. (**d**) The percentage of Bax/Bcl-2 ratio was presented in the bar graphs

To further confirm the induction of apoptosis in HT29 and CT26 cells with nifuroxazide treatment, we also investigated the levels of apoptosis by Annexin V-FITC/PI dual-labeling technique by flow cytometry (FCM). As shown in Figures 2a and b, after nifuroxazide treatment for 24 h, the apoptosis induction effects were observed. When the CT26 cells were treated with 5 *μ*M nifuroxazide, the apoptosis rate was 6.5%, whereas the apoptosis cells increased to 9.3 and 33.2% when cells were treated with 10 and 20 *μ*M nifuroxazide, respectively, indicating that nifuroxazide was able to induce apoptosis in a concentration-dependent manner. Similarly, in HT29 cells, the percentage of apoptotic cells was increased from 4.5 to 13.85% and 60.1% after treatment with various concentration of nifuroxazide for 24 h. The above results implied that the inhibition of CRC cells by nifuroxazide is mediated by the induction of apoptosis.

### Nifuroxazide-induced apoptosis via the mitochondria-mediated apoptotic pathway

To further confirm that nifuroxazide-induced tumor cell death was associated with apoptosis, we analyzed the levels of Bcl-2, Bax and cleaved caspase-3 in CT26 cells treated with nifuroxazide using western blot. As shown in Figures 2c and d, the expression of Bcl-2 was significantly reduced, whereas that of Bax and cleaved caspase-3 increased in a concentration-dependent manner, and a significant increase in the ratio of Bax/Bcl-2 was also seen (Figure 2d), suggesting that nifuroxazide-induced apoptosis might be via the mitochondrial apoptotic pathway.

**Figure 3:**
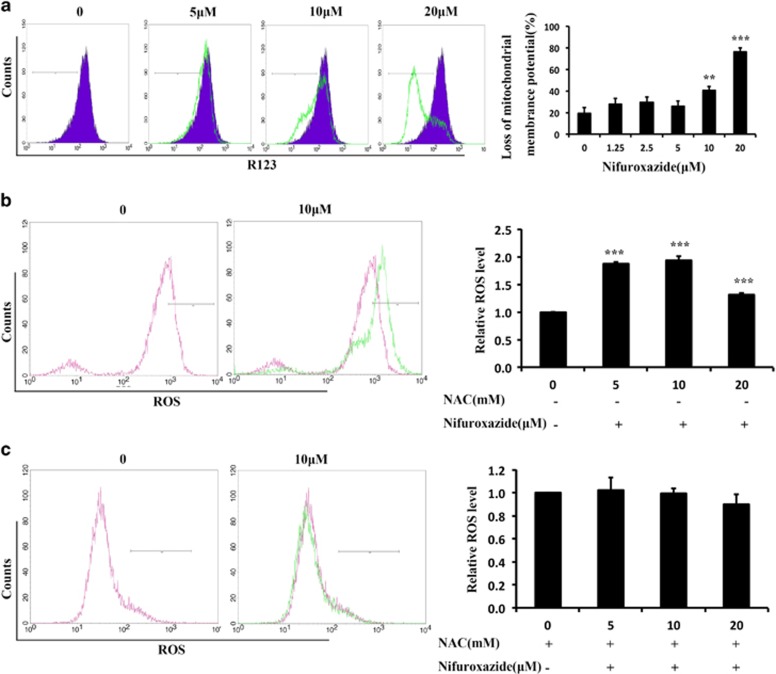
Effects of nifuroxazide on the intrinsic apoptosis pathway. (**a**) Nifuroxazide decreased the mitochondrial membrane potential (ΔΨ_m_) in CT26 cells. CT26 cells were treated with different concentrations of nifuroxazide for 24 h and then stained with 5 *μ*g/ml Rh123 to test the changes of ΔΨ_m_ by FCM. The quantified values are also shown. (**b** and **c**) The levels of ROS were increased after treatment with nifuroxazide. CT26 cells were pre-treated or not pre-treated with with 2 mM NAC for 1 h and then treated with 0–20 *μ*M nifuroxazide. The harvested cells were incubated with 10 *μ*M DCFH-DA for 30 min at 37 °C and measured by FCM. Data are expressed as mean±S.D. for at least three independent experiments (**P<0.01 and ***P<0.001 compared with the vehicle group)

To verify the hypothesis, we assessed the changes in the mitochondrial membrane potential (ΔΨ_m_) using a green fluorochrome Rh123 (2-(6-amino-3-imino-3*H*-xanthen-9-yl) benzoic acid methyl ester) by FCM. As shown in Figure 3a, a significant loss of ΔΨ_m_ was observed after nifuroxazide treatment. Moreover, the mitochondria are the major source of ROS generation. In the present study, ROS formation was detected by FCM using an indicator, DCFH-DA. The results showed that the levels of ROS increased after treatment with nifuroxazide (Figure 3b). However, the increased ROS levels were attenuated by treatment with the antioxidant *N*-acetyl-l-cysteine (NAC) for 1 h (Figure 3c). These results confirmed that the inhibition of CRC cells by nifuroxazide is mediated by the induction of apoptosis through the mitochondria-mediated apoptotic pathway.

### Nifuroxazide impaired cellular migration and invasion

**Figure 4:**
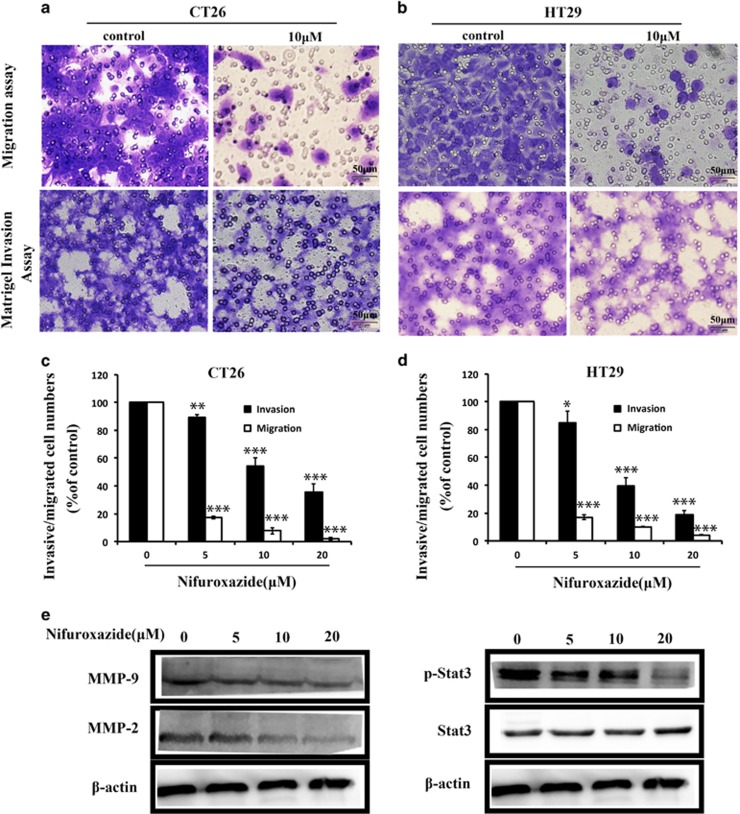
Nifuroxazide inhibited CT26 and HT29 cells migration and invasion. (**a** and **b**) Nifuroxazide inhibited the migration of CT26 and HT29 cells in the Transwell assay. In the Transwell assay, cells were seeded in the top chamber of Transwell with serum-free medium and treated with various concentrations of nifuroxazide. After 24 h, the migrated cells were fixed, stained and quantified. Nifuroxazide inhibited the invasion of CRC cells in Transwell assay. A total of 1 × 10^5^ cells were planted, which were pre-treated with Matrigel on the upper chamber membrane and treated with indicated concentrations of nifuroxazide, and the bottom chamber was filled with the complete medium containing 10% FBS. (**c**) Statistical results of migration and invasion assays of CT26 cells. Invaded cell number was counted (*P<0.05, **P<0.01 and ***P<0.001). (**d**) Statistical results of migration and invasion assays of HT29 cells. Invaded cell number was counted (*P<0.05, **P<0.01 and ***P<0.001). (**e**) Western blot analysis of CT26 cells following nifuroxazide treatment, including the expressions of Stat3/p-Stat3, MMP-2 and MMP-9; *β*-actin served as a loading control

Transwell assays were used to assess the effects of nifuroxazide on cell migration and invasion. As shown in Figures 4a and b, the Transwell migration assay indicated that nifuroxazide significantly inhibited the migration of both HT29 and CT26 cells in a dose-dependent manner (Figures 4c and d). Moreover, Transwell invasion assays assessed the ability of HT29 and CT26 cells to invade through the Matrigel. As shown in Figures 4a and b, both HT29 and CT26 cells exhibit significantly decreased invasion in the presence of nifuroxazide compared with that of vehicle (Figures 4c and d). To further confirm the effects of nifuroxazide on cell migration and invasion, we investigated whether p-Stat3^Tyr705^, MMP-2 and MMP-9, which are considered to be associated with cell migration and invasion,^29^ are involved in nifuroxazide-mediated cell migration and invasion. The results of western blot indicated that the treatment with nifuroxazide significantly inhibited the expression of p-Stat3, MMP-2 and MMP-9 without affecting the total Stat3 expression level in CT26 cells (Figure 4e). Altogether, all of our results implied that nifuroxazide inhibited the migration and invasion of CRC cells *in vitro*.

### Antimetastasis efficacy of nifuroxazide *in vivo*

**Figure 5:**
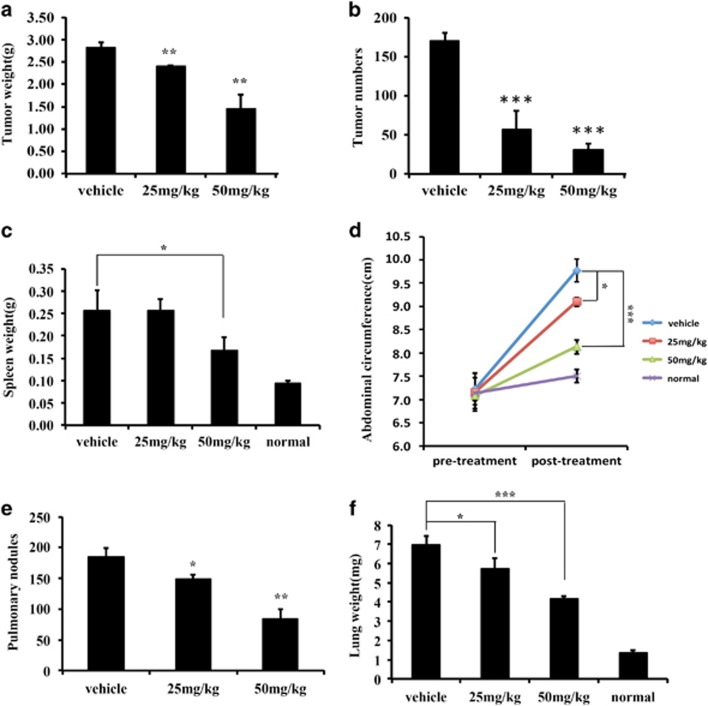
Antimetastasis efficacy of nifuroxazide *in vivo.* To establish an abdominal metastasis model, a total of 5 × 10^5^ CT26 cells were intraperitoneally injected. On day 6 after post-tumor inoculation, mice were treated with 25 and 50 mg/kg per day of nifuroxazide (*P<0.05, **P<0.01 and ***P<0.001 *versus* vehicle control). (**a**) Weight of tumor nodules in abdomen metastasis model. (**b**) Metastatic tumor nodule numbers in abdomen metastasis model. (**c**) Weight of spleen in abdomen metastasis model. (**d**) AC was calculated before and after treatment. To establish a pulmonary metastasis model, a total of 5 × 10^5^ CT26 cells were injected into the tail vein of syngeneic Balb/C mice. On day 6 after post-tumor inoculation, mice were treated with 25 and 50 mg/kg per day of nifuroxazide (*P<0.05, **P<0.01 and ***P<0.001 *versus* vehicle control). (**e**) Metastatic tumor nodules number in lung metastasis model. (**f**) Weight of lung in lung metastasis model

To investigate whether the antitumor activity of nifuroxazide *in vivo* is consistent with its effects *in vitro*, two tumor-bearing mice models were established. CT26 cells were injected into Balb/C mice intravenously to establish the lung metastasis model and intraperitoneally to establish the abdominal metastasis model. The mice received the following treatments: vehicle, nifuroxazide at 25 mg/kg and nifuroxazide at 50 mg/kg. In the abdominal implantation model, the abdominal circumference (AC) was calculated from the abdominal anteroposterior diameter before and after treatment, the tumor in the abdominal cavity were removed and weighed, and tumor nodules were counted. As shown in Figures 5a and b, the administration of nifuroxazide resulted in a significant reduction in the weights of tumor nodules and a marked reduction in the number of tumor nodules compared with that of the negative group. In addition, nifuroxazide-treated group significantly reduced splenomegaly compared with control groups (Figure 5c). Furthermore, there was a significant reduction in AC of mice treated by nifuroxazide compared with the vehicle group.

In the lung metastasis model, the lungs were removed, weighed and counted the total number of lung metastatic nodules under the dissection scope. As shown in Figure 5e, the number of lung metastatic nodules was significantly decreased in the nifuroxazide-treated group mice. Moreover, nifuroxazide-treated group had a >1.8-fold reduction in the weights of the lungs compared with the negative vehicle group. Besides, when given nifuroxazide, it had a ~1.5-fold reduction in the number of metastatic nodules on the lungs compared with the vehicle group (Figure 5f). Overall, these results indicated that the treatment with nifuroxazide almost fully prevented lung metastases.

### Nifuroxazide modulated the metastatic environment in the abdominal metastasis model

**Figure 6:**
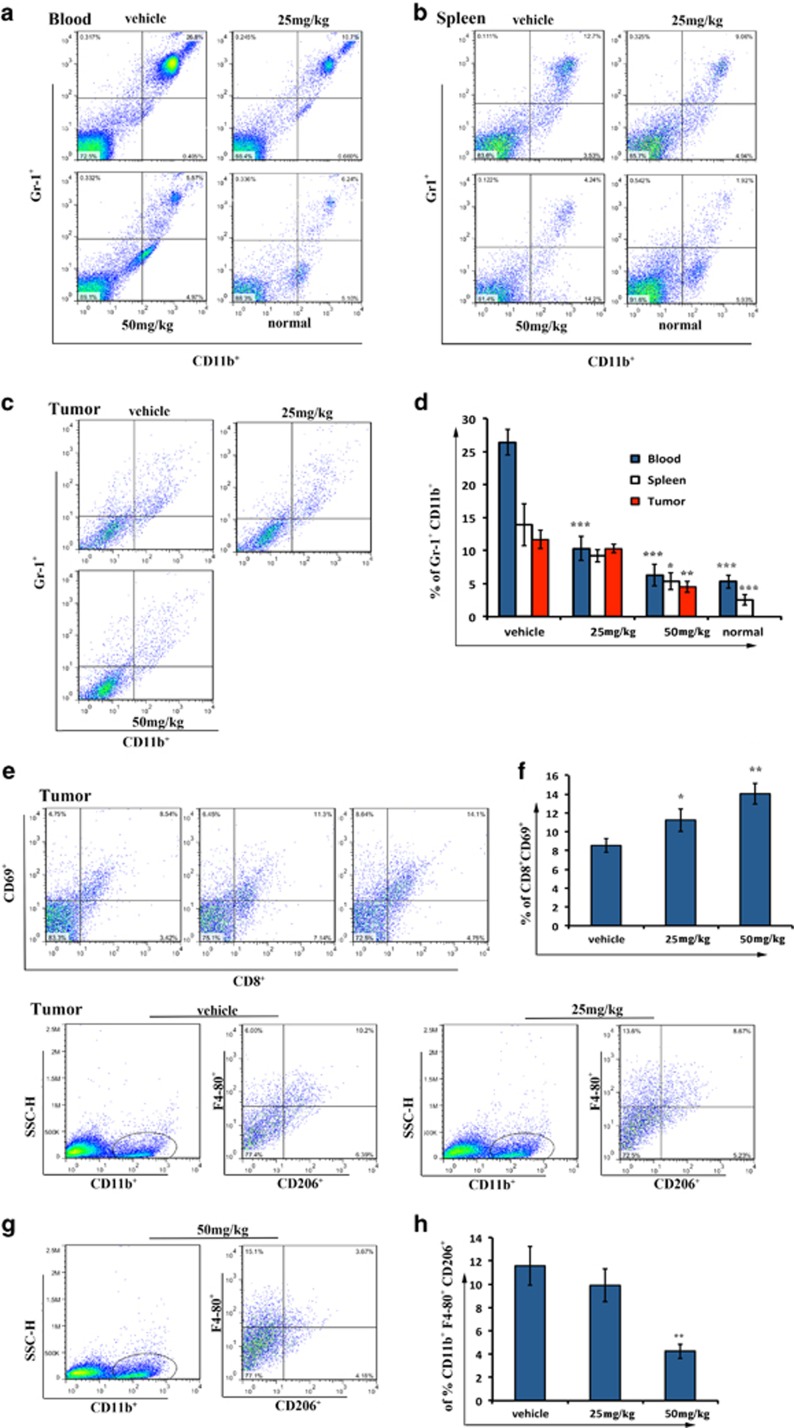
Nifuroxazide modulated immune cells in the abdominal metastasis model. (**a**–**c**) Nifuroxazide significantly reduced tumor-associated MDSCs in the abdominal metastasis model. Flow cytometry analysis quantified CD11b+Gr1+ myeloid cells in peripheral blood, spleen and tumors after treatment with nifuroxazide. (**d**) Statistic results of the abdominal metastasis model. Bars show mean±S.D.; five mice every group (*P<0.05, **P<0.01 and ***P<0.001). (**e** and **f**) Single-cell suspensions prepared from tumors were analyzed by flow cytometry for the presence of CD8+CD69+. Bars show mean±S.D.; five mice every group (*P<0.05 and **P<0.01). (**g** and **h**) M2-type macrophages from tumors were analyzed by flow cytometry for the presence of CD11b+F4/80+CD206+. Bars show mean±S.D.; three mice every group (**P<0.01)

Previous studies have shown Stat3 as an important molecule in MDSC accumulation in tumor-bearing mice. Moreover, it has been shown that accumulation of MDSCs has a key role in the development of metastasis.^18^ Therefore, we examined the number of MDSCs in organs and tumors in the abdominal implantation metastasis model by FCM with CD11b and Gr1 antibody. As shown in Figures 6a and b, the number of CD11b^+^/Gr1^+^ MDSCs were significantly decreased in both spleens and peripheral blood after treatment with nifuroxazide compared with vehicle group. In particular, we observed a fourfold reduction in blood infiltration MDSCs in nifuroxazide-treated groups compared with untreated groups (*P*<0.001). Moreover, we further examined tumor-associated MDSCs infiltration, and from the FCM data, we found ~2.5-fold reduction in MDSCs in the tumor after nifuroxazide treatment (Figure 6c). Furthermore, the tumor infiltration of active CD8^+^ T lymphocytes was increased in the nifuroxazide-treated group compared with the control group (*P*<0.01) (Figures 6e and f).

Tumor-associated macrophages are important immune cells that exist in tumor microenvironments and have a role in modulating tumor-associated immune reaction. Moreover, the M2-type macrophages usually have a negative role in anticancer therapy by releasing cytokines that can promote cancer progression.^30^ Therefore, we tested the number of M2-type macrophages in tumor by FCM with the surface marker CD11b^+^F4/80^+^CD206^+^. As shown in Figures 6g and h, the number of M2-type macrophages were significantly decreased in tumor after treatment with nifuroxazide compared with the vehicle group.

### The lung metastatic environment was modulated by nifuroxazide in the lung metastasis model

**Figure 7:**
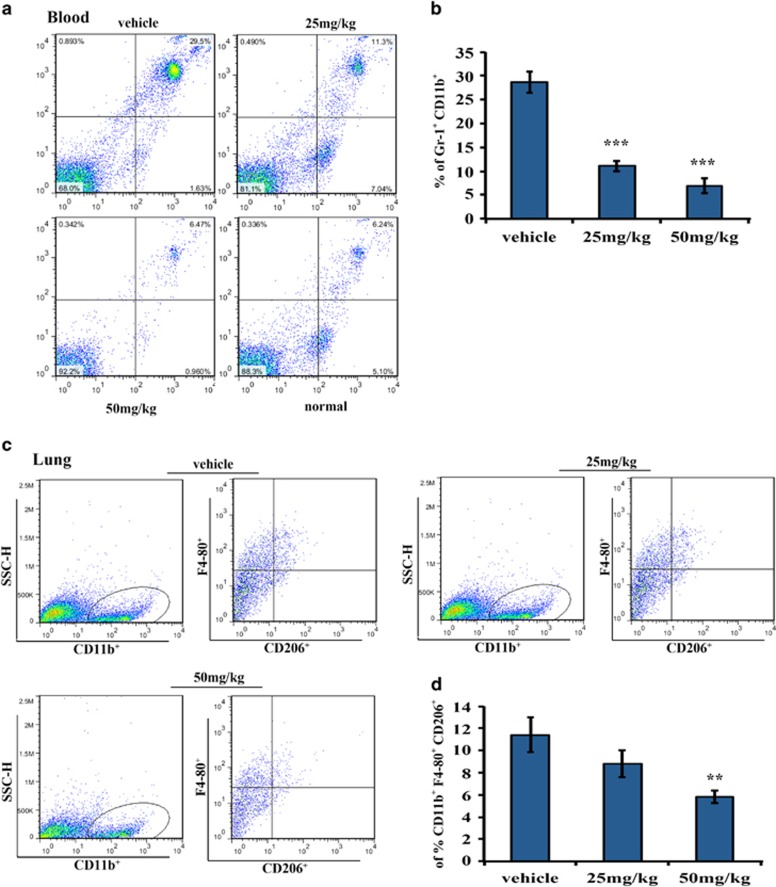
Nifuroxazide modulated MDSCs and M2-type macrophages in the lung metastasis model. (**a** and **b**) Nifuroxazide significantly reduced tumor-associated MDSCs in the lung metastasis model. Flow cytometry analysis quantified CD11b+Gr1+ myeloid cells in peripheral blood after treatment with nifuroxazide. Statistic results of the lung metastasis model. Bars show mean±S.D.; five mice every group (**P<0.01 and ***P<0.001). (**c** and **d**) M2-type macrophages from lung were analyzed by flow cytometry for the presence of CD11b+F4/80+CD206+. Bars show mean±S.D.; three mice every group (**P<0.01)

To further confirm that nifuroxazide could inhibit tumor metastasis, in part, by affecting the tumor microenvironment, we also investigated the changes of immune cells in the lung metastasis model. As shown in Figures 7a and b, we observed ~4-fold reduction of MDSCs in nifuroxazide-treated groups compared with vehicle groups in the blood. However, statistical analysis also demonstrated that nifuroxazide did not significantly decrease the number of MDSC in the spleen and lung compared with that of the vehicle group (data not shown). Then, we determined whether nifuroxazide treatment could reduce the presence of M2-type macrophage cells in the lungs. As shown in Figures 7c and d, the number of M2-type macrophages were significantly decreased in the lungs after treatment with nifuroxazide was observed.

### Nifuroxazide inhibited proliferation, induced apoptosis and impaired metastasis *in vivo*

**Figure 8:**
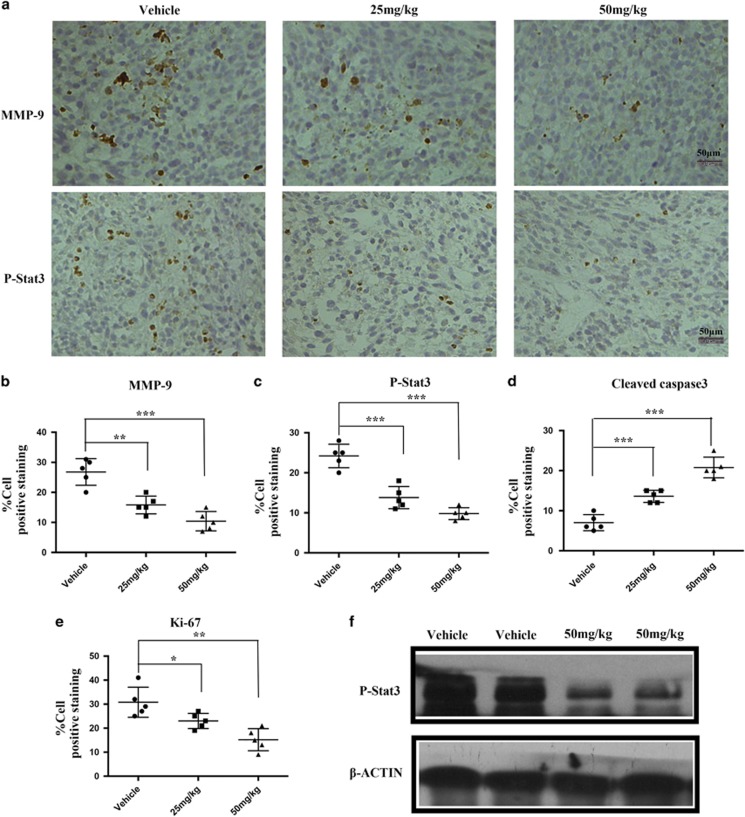
Nifuroxazide reduced tumor cell proliferation, and induced tumor apoptosis in the abdominal metastasis model. To establish an abdominal metastasis model, a total of 5 × 10^5^ CT26 cells were intraperitoneally injected. On day 6 after post-tumor inoculation, mice were treated with 25 and 50 mg/kg per day of nifuroxazide (*P<0.05, **P<0.01 and ***P<0.001 *versus* vehicle control). (**a** and **b**) The immunohistochemical analysis was performed to measure the expressions of MMP-9 in tumor tissues. *P*-values for comparison of two groups were determined by TWO-tailed Student's *t*-test. (**a** and **c**) The immunohistochemical analysis was performed to measure the expressions of P-Stat3 in tumor tissues. *P*-values for comparison of two groups were determined by TWO-tailed Student's *t*-test. (**d**) The statistical data of cleaved caspase-3-positive cell number were shown. (**e**) The statistical data of Ki-67-positive cell number were shown. (**f**) The expression of p-Stat3 in the tumor treated or not treated by nifuroxazide by western blot

To define the mechanisms through which nifuroxazide elicited CRC tumor growth inhibition *in vivo*, immunohistochemistry (IHC) and the western blot analyses were performed in tumor tissues. There is increasing evidence to indicate that MMPs have important roles in tumor invasion and metastasis.^29^ We therefore measured the effects of nifuroxazide on the expression of MMP-9 by IHC; as shown in Figures 8a and b, treatment of mice with nifuroxazide inhibited the expression of MMP-9 in CT26 tumor tissues. Moreover, we also found that treatment with nifuroxazide could inhibit the expression of p-Stat3 in CT26 tumor tissues (Figures 8a and c). Furthermore, western blot analyses of the whole tumor lysates revealed a marked inhibition of p-Stat3 by nifuroxazide treatment (Figure 8f). These results suggest that nifuroxazide has significant antitumor effects *in vivo*, with at least inhibition of Stat3 signaling pathway. In addition, nifuroxazide significantly induced apoptosis cells of cleaved caspase-3-positive cells and inhibited the proliferation of nuclear Ki-67-positive cells (Figures 8d and e). Our data demonstrated that nifuroxazide could inhibit/reverse early metastasis in CRC, which is consistent with the *in vitro* data.

## Discussion

CRC is one of the most common cancers worldwide with considerable metastatic potential and drug resistance, and the incidence is increasing rapidly. Diverse therapies such as radiation, chemotherapy and immunotherapy have shown beneficial effects, but are limited because of their safety and toxicity.^31^ There is an urgent need to discover the novel potential drug candidate against CRC. Recent studies have reported constitutive activation of Stat3 signaling pathway in human CRCa. Besides, the level of activated p-Stat3 increased in 45 primary CRC samples compared with adjacent normal mucosae has been reported.^6^ Therefore, directly inhibiting Stat3 might be a promising novel approach for CRC therapy.

In the present study, nifuroxazide, an oral antidiarrheal agent identified as an inhibitor of Stat3, was evaluated for the first time for its potency against CRC *in vitro* and *in vivo*. Our results showed that three CRC cell lines were sensitive to nifuroxazide. In addition, nifuroxazide could inhibit CRC cell viability in a time- and dose-dependent manner, and clonogenicity assay was performed to visually confirm this result. Moreover, in our established abdomen metastasis model, fewer cells that were Ki-67-positive were observed in the tumor tissues treated with nifuroxazide than in the untreated group, indicating that nifuroxazide could inhibit cell proliferation both *in vitro* and *in vivo*.

Apoptosis is a major route to eradicate cancer cells and is controlled by a diverse range of cell signals.^32^ In the intrinsic apoptosis pathway, the mitochondria have an important role by altering the mitochondrial transmembrane potential. This apoptosis pathway involves the participation of Bcl-2 family proteins, including the proapoptotic protein Bax and the antiapoptotic protein Bcl-2.^33^ In this study, Hoechst 33358 staining and FCM assays both revealed that nifuroxazide treatment induced apoptosis on CRC cells in a concentration-dependent manner. Moreover, the activation of CC-3 was observed after treatment with nifuroxazide both *in vitro* and *in vivo*. Importantly, the occurrence of apoptosis was associated with the activation of Bax and the downregulation of Bcl-2, which also induced a loss of ΔΨ_m_ in CT26 cells after nifuroxazide treatment. Furthermore, many studies have shown that disruption of the ROS homeostasis has an important role in mitochondrial dysfunction and apoptotic events;^34, 35^ we also found that nifuroxazide significantly increased ROS production in CT26 cells, while the enhanced ROS levels were attenuated by pretreatment with antioxidant NAC, which indicated that the effects of nifuroxazide in CT26 cells may be associated with cell redox system imbalance. Therefore, these results indicated that nifuroxazide treatment induced apoptotic death on CRC through ROS-medicated mitochondrial apoptotic pathway. However, several questions remain: Why were the levels of ROS lower when drug dose was increased (20 *μ*M compared with 10 *μ*M)? Once ROS levels reach a certain degree, is increasing the drug dose helpful for ROS production? Else, does this lead to other side effects and reduce chemotherapy tolerance? Therefore, in-depth studies are still needed to explain this phenomenon.

It has been reported that ~30–50% of CRC patients who undergo curative resection subsequently experience local tumor recurrence or metastasis.^36^ Moreover, tumor cell migration and invasion is a key step in successful cancer metastasis, and inhibition of this important step is a practical approach to antitumor treatment.^37, 38^ Therefore, the inhibitory effects of nifuroxazide on metastasis were evaluated by cell invasion and migration assays. The Transwell assays indicated that nifuroxazide inhibited HT29 and CT26 cell migration and invasion. Moreover, some cell invasion- and migration-related proteins, such as p-Stat3^Tyr705^, MMP-2 and MMP-9, were downregulated by nifuroxazide in CT26 cells. Similar results were observed in the abdominal implantation model, with reduction in the number of MMP-9- and p-Stat3^Tyr705^-positive metastasis cells, as analyzed by IHC and western blot. Then, we used the lung and abdomen metastasis models of colon cancer to further verify this result. The number of lung metastatic nodules, the lung weight and the tumor nodules in the two metastasis models also indicated that nifuroxazide prevented the generation of lung and abdomen metastases.

Recently published studies showed that a complex multidirectional interaction exists between tumor cells surrounding the stroma and the microenvironment at metastatic sites.^39, 40^ Moreover, infiltration and accumulation of tumor-associated myeloid cells into the lungs and other organs have a crucial role in the development of metastasis.^41^ In addition, Stat3 is constitutively activated not only in tumor cells but also in tumor endothelial and myeloid cells, including MDSCs and tumor-associated macrophages,^17, 42, 43^ promoting the expression of a large number of metastatic and angiogenic factors. Therefore, Stat3 is important for tumor angiogenesis and metastasis.^12, 18, 44^ Our *in vivo* studies indicated that the treatment of mice with nifuroxazide caused a significant reduction in the number of MDSCs in the spleen, blood and tumors compared with that of the vehicle group, which was also accompanied by an increased infiltration of CD8^+^ T cells in the tumors. Importantly, nifuroxazide markedly inhibited the number of M2-type macrophages in tumor in the abdomen metastasis model. It is therefore conceivable that nifuroxazide could potentiate the antimetastasis effects by stimulating the antitumor immune responses. However, in the lung metastasis model, nifuroxazide did not significantly decrease the number of MDSC in the spleen and lung compared with that of the vehicle group. We doubt that the modest lung metastasis *in vivo* might be due to the low dose of nifuroxazide and its pool bioavailability. Therefore, more investigations will be needed to enhance its antimetastatic activities *in vivo*.

In conclusion, our present studies provide important information regarding the antimetastatic activities of nifuroxazide in CRC. To our knowledge, this is the first study to demonstrate that the anti-CRC activities of nifuroxazide *in vitro* and *in vivo*. Mechanism studies showed that nifuroxazide could inhibit cancer cell growth and cell apoptosis via ROS-mitochondrial apoptotic pathway. Moreover, we further found that nifuroxazide markedly blocked cell migration and invasion. Importantly, nifuroxazide could inhibit metastasis by reducing the number of MDSCs and M2-type macrophages in tissues and enhancing the antitumor immune responses. Our current study further implied that Stat3 activity is important for CRC response to nifuroxazide, and Stat3 inhibition by nifuroxazide direct the proapoptotic activity on tumor cells and positive effects on tumor immunologic microenvironment. Taken together, blocking Stat3 signaling pathway with nifuroxazide may, in part, have promise in the treatment of CRC by inhibiting invasion and metastasis.

## Materials and Methods

### Regents and preparation of nifuroxazide

Nifuroxazide was purchased from Xiyashiji Chemical Co. Ltd (Chengdu, Sichuan, China), and was measured by ^1^H-NMR, ^13^C-NMR and ESI-MS analysis. For the *in vitro* assays, nifuroxazide was prepared in dimethyl sulfoxide (DMSO) at a stock concentration of 20 mM and stored at −20 °C. Then, it was diluted in the relevant medium at a final DMSO concentration of 0.1% (v/v), and the medium with 0.1% DMSO served as a vehicle control. For *in vivo* studies, nifuroxazide was dissolved in 25% (v/v) Cremophor EL/ethanol (50 : 50, Sigma Cremophor EL, 100% ethyl alcohol) and 75% ultrapure water.

DMSO, MTT, DCFH-DA, Rh123 and Cremophor EL were from Sigma Chemical Co. (St. Louis, MO, USA). Hoechst 33258 and NAC were obtained from Beyotime (Beijing, China). The primary antibodies against Stat3/p-Stat3^Tyr705^, MMP-2, MMP-9, cleaved caspase-3, Bax, Bcl-2 and *β*-actin were purchased from Cell Signaling Technology (Beverly, MA, USA). FITC-CD11b-, PE-Gr1-, FITC-CD8a-, PE-CD69-, PE-CD206- and APC-F4/80-conjugated antibodies were obtained from BD Biosciences (San Diego, CA, USA). Anti-Ki-67 mouse monoclonal was purchased from Merck Millipore (Billerica, MA, USA). The Annexin V-FITC Apoptosis Detection Kit was obtained from KeyGen Biotech (Nanjing, China).

### Cell culture

Human CRCa cell lines, HT29 and HCT116, and the mouse CRCa cell line, CT26, were purchased from the American Type Culture Collection (Rockville, MD, USA). All of them were propagated in DMEM or RPMI-1640 media supplemented with 10% fetal bovine serum (FBS; Gibco, Auckland, New Zealand) and 1% antibiotics (penicillin and streptomycin) in 5% CO_2_ at 37 °C.

### Cell proliferation assay

The cell viability of nifuroxazide-treated cancer cells was determined using the MTT assay. In brief, cells (3–5 × 10^3^cells per well) were seeded in 96-well culture plates. After 24 h incubation, the cells were treated with different concentrations of nifuroxazide. After being treated for 24, 48 or 72 h, respectively, the 20 *μ*l of a 5 mg/ml MTT solution was added to each well and incubated for an additional 2–4 h at 37 °C. The medium was subsequently removed, and 150 *μ*l DMSO was added. Absorbance was measured at 570 nm using a Spectra MAX M5 microplate spectrophotometer (Molecular Devices, Sunnyvale, CA, USA), and the inhibition of cell lines was detected. Each experiment was repeated at least three times.

### Colony formation assay

Briefly, cells were assayed for the colony-forming ability by replating them in specified numbers (400–600 cells per well) and were seeded in a 6-well plate. After 24 h incubation, the cells were treated with various concentrations of nifuroxazide and then cultured for another 12 days. Finally, the cells were washed with cold PBS, colonies were fixed with 4% paraformaldehyde and stained with a 0.5% crystal violet solution for 15 min, and the colonies (>50 cells) were counted under a microscope.

### Morphological analysis by Hoechst staining

To identify the apoptosis induction effects of nifuroxazide, we analyzed the morphological changes associated with apoptosis by Hoechst 33358 staining. After incubating with nifuroxazide for 24 h, the cells were washed with PBS two times and fixed with 4% paraformaldehyde and stained with Hoechst 33358 solutions (5 *μ*g/ml). Then, the nuclear morphology of the cells was observed by fluorescence microscopy (Leica DM4000B; Leica, Wetzlar, Germany).

### Apoptotic assays

To further investigate the apoptosis induction effects of nifuroxazide, apoptotic cells were detected by FCM. Briefly, cells (1–2 × 10^5^ cells per well) were seeded in a 6-well plate overnight and treated with nifuroxazide. After 24 h of treatment, the cells were harvested and washed with PBS. The levels of apoptosis were examined using an Annexin V-FITC Apoptosis Detection Kit according to the manufacturer's instructions by FCM (BD Biosciences). Data were analyzed using the FlowJo software (Tree Star, San Carlos, CA, USA).

### Measurement of ROS levels in cells

After exposure to different concentrations of nifuroxazide for 24 h, CT26 cells were incubated with DCFH-DA (10 *μ*M) at 37 °C for ~20 min. The fluorescence intensity was measured by FCM. To further detect the changes in ROS, antioxidant treatment by 2 mM NAC (an ROS inhibitor) for 1 h was carried out before nifuroxazide exposure.

### ΔΨ_m_ assay

Rh123 was used to test the changes in ΔΨ_m_ by FCM. In brief, cells (1–2 × 10^5^ cells per well) were seeded in a 6-well plate overnight and treated with nifuroxazide for 24 h, and then the harvested cells were washed with cold PBS and incubated with Rh123 solution (5 *μ*g/ml) at 37 °C for 30 min in the dark and then ΔΨ_m_ was measured by FCM.

### Boyden chamber migration and invasion assay

Modified Transwell invasion assay was conducted as described previously with minor modifications.^26, 27^ Briefly, Matrigel (BD Biosciences) diluted 1 : 3 in serum-free medium was added (60 *μ*l per well) to the upper surface of 24-well Transwell plate (Millipore, Cambridge, MA, USA). After Matrigel polymerization, the top of the Matrigel layer was seeded with 1 × 10^5^ CT26 or HT29 cells in 100 *μ*l serum-free medium to which with 0.1% DMSO or various concentrations of nifuroxazide were added, and the lower compartments were filled with 600 *μ*l of the complete medium, which served as a chemoattractant. After 24 h, the cells on the top were moved with a cotton swab, and the invasion cells on the filters were stained with crystal violet after being fixed with 4% paraformaldehyde. The images were taken using a Zeiss digital microscope (Zeiss, Jena, Germany). Five independent areas per well were counted and the mean number of migrated cells was calculated. Boyden chamber migration assay was performed according to the previous studies. A total of 1 × 10^5^ CT26 or HT29 cells in 100 *μ*l serum-free medium were added to the upper chamber, and the bottom chamber was filled with 600 *μ*l of the complete medium containing 10% FBS. Both chambers were added with different concentrations of nifuroxazide. After 24 h, a cotton swab was used to discard the non-migrated cells in the upper chamber, and the migrated cells were stained with 0.5% crystal violet. In total, six random fields were counted and photographed under a light microscope.

### Flow cytometry

At the indicated time points, we prepared single-cell suspensions of tumor, spleen, lung and blood by mechanic and enzymatic dispersion as described previously.^45^ Then, 1 × 10^6^ freshly prepared cells were suspended in 100 *μ*l PBS and stained with different combination of fluorochrome-coupled antibodies to F4/80, CD206, CD11b, CD8, CD69 and Gr1. Cells were collected by FCM and data were analyzed using the FlowJo software.

### Western blot assay

Western blot analysis was performed as described previously, with some modification.^26, 27^ Briefly, CT26 cells were treated with nifuroxazide in different concentrations for 24 h. Harvested cells were washed two times with ice-cold PBS and lysed in RIPA buffer. Protein concentrations were examined using the Lowry method and equalized before loading. The cell samples or tumor lysates were applied to SDS-PAGE gels and transferred onto polyvinylidene difluoride membranes (Amersham Bioscience, Piscataway, NJ, USA). Next, the membranes were incubated with specific primary antibodies overnight at 4 °C, followed by horseradish peroxidase-conjugated secondary antibodies. After being incubated with secondary antibodies, the reactive bands were identified using a commercially available Enhanced Chemiluminescence Kit (Amersham Bioscience).

### Mice, tumor model and treatment

The mice models were as described previously.^46^ To establish a pulmonary metastasis model, a total of 5 × 10^5^ CT26 cells were injected into the tail vein of Balb/C mice. To establish an abdominal metastasis model, a total of 5 × 10^5^ CT26 cells were intraperitoneally injected. On day 6 after post-tumor inoculation, each model was randomized into three groups with eight mice in each group for further administration as follows: vehicle, group of 25 mg/kg and group of 50 mg/kg. In the abdominal metastasis model, AC was calculated before and after treatment of nifuroxazide. The animals were killed on day 12 after the first treatment. Seeding metastatic tumor nodules in the abdominal cavity were counted by naked eyes and weighted. Metastatic tumor nodules in the subpleural regions of the lung were counted under a dissecting microscope and weighted.

### Immunohistochemistry

IHC staining of tumors sections was described previously.^27^ Paraffin-embedded tumor sections were stained with primary antibodies (MMP-9, p-Stat3, cleaved caspase-3 and Ki-67) using the DAB detection Kit (ZSGB-BIO Co., Beijing, China).

### Statistical analysis

All experiments were performed at least in triplicate. Results are expressed as mean±S.D. and *P*-values for comparison of two groups were determined by two-tailed Student's *t*-test. Statistically significant *P*-values were labeled as follows: *P<0.05, **P<0.01 and ***P<0.001.
