# Correction: Inhibition of Stat3 signaling pathway by nifuroxazide improves antitumor immunity and impairs colorectal carcinoma metastasis

**DOI:** 10.1038/s41419-019-1955-9

**Published:** 2019-09-26

**Authors:** Ting-Hong Ye, Fang-Fang Yang, Yong-Xia Zhu, Ya-Li Li, Qian Lei, Xue-Jiao Song, Yong Xia, Ying Xiong, Li-Dan Zhang, Ning-Yu Wang, Li-Feng Zhao, Hong-Feng Gou, Yong-Mei Xie, Sheng-Yong Yang, Luo-Ting Yu, Li Yang, Yu-Quan Wei

**Affiliations:** 1 Department of Liver Surgery and Division of Digestive Diseases, State Key Laboratory of Biotherapy/Collaborative Innovation Center for Biotherapy, West China Hospital, West China Medical School, Sichuan University, Chengdu, China; 2 Department of Pharmacy, Xinqiao Hospital, Third Military Medical University, Chongqing, China; 3 Department of Abdominal Cancer, Cancer Center, West China Hospital, West China Medical School, Sichuan University, Chengdu, China

**Correction to: Cell Death & Disease**

10.1038/cddis.2016.452 published online 5 January 2017

Since publication of this article, the authors have noticed that there were errors in Fig. 1b (the CT 26 cells colony formation images) and Fig. 7c (the vehicle group images). As a result of the misfiling of the data during preparation of figures, incorrect images were inadvertently inserted in these figures. The correct figures are given below. Our corrections do not affect the original conclusions of this paper. The authors would like to apologize for any inconvenience caused.

**Figure:**
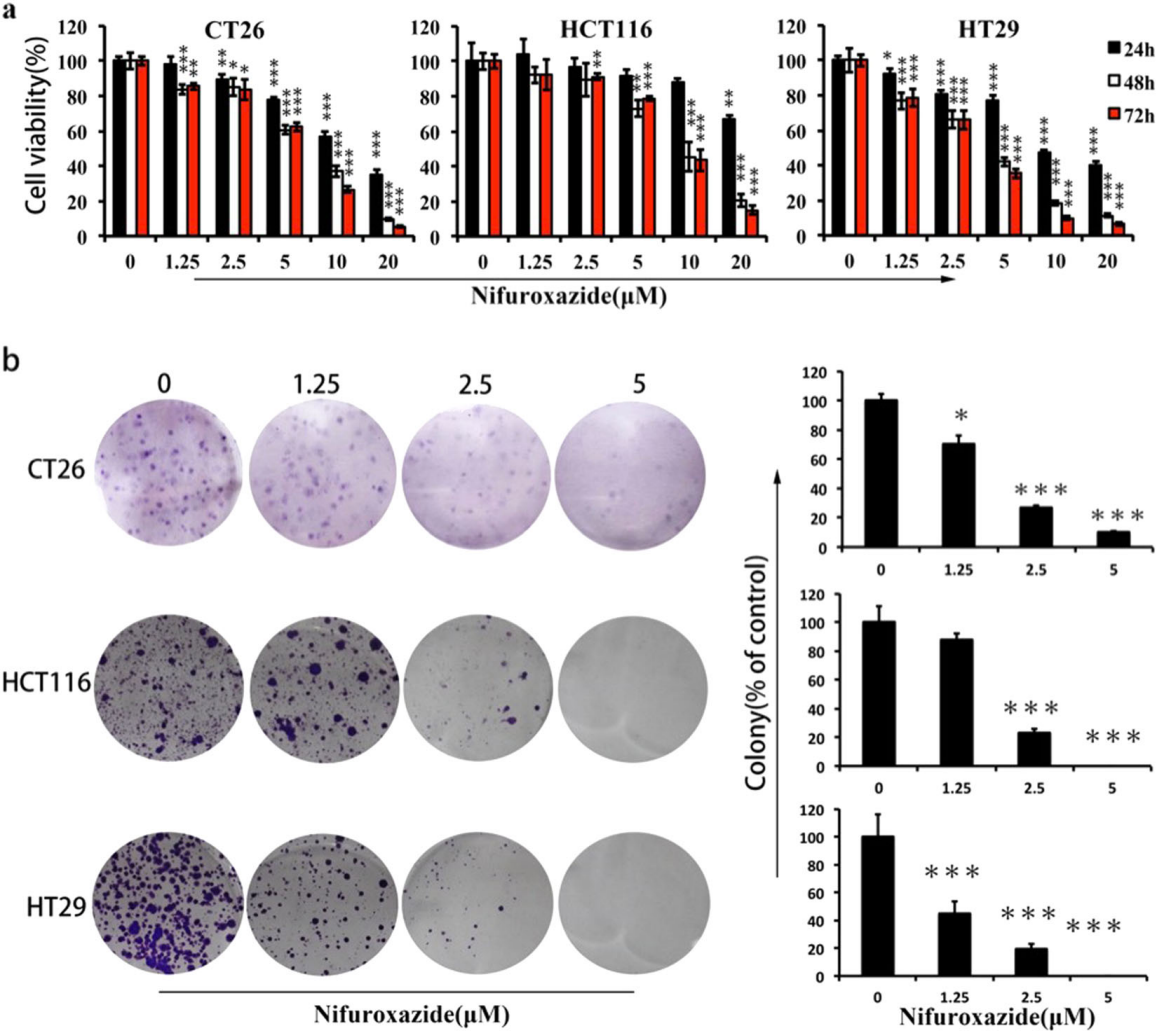
Fig. 1

**Figure:**
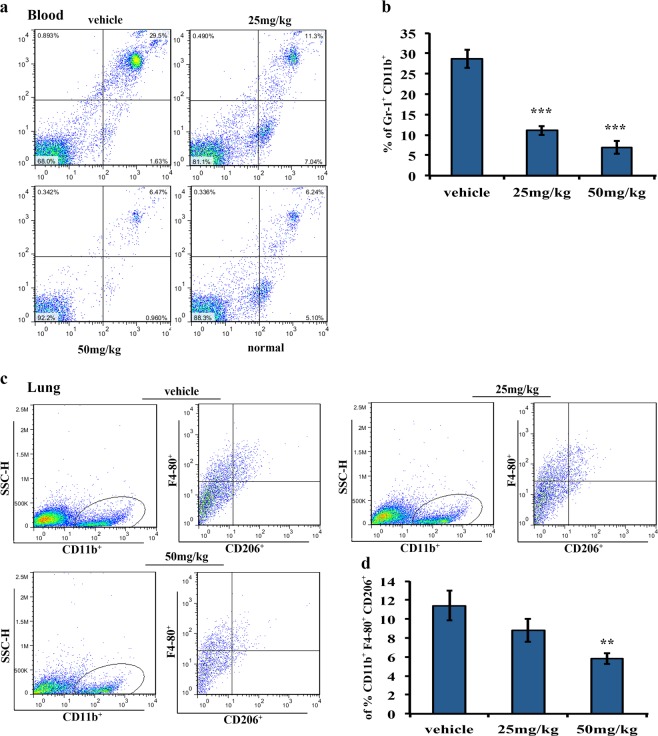
Fig. 7

