# Supplementary material for: Inhibition of Stat3 signaling pathway by nifuroxazide improves antitumor immunity and impairs colorectal carcinoma metastasis
Source: Cell Death Dis. 2017 Jan 5;8(1):e2534–. doi: 10.1038/cddis.2016.452 (PMC5386364; doi:10.1038/cddis.2016.452)

**Inhibition of** **Stat3 signaling pathway by nifuroxazide improves antitumor immunity and impairs colorectal carcinoma metastasis**

**Running Title :** **Anti-colon cancer activity of Nifuroxazide**

Tinghong Ye^1,#^, Fangfang Yang^1,#^, Yongxia Zhu^1^, Yali Li^1^, Qian Lei^1^, Xuejiao Song^1^, Yong Xia^1^, Ying Xiong^1,2^, Lidan Zhang^1^, Ningyu Wang^1^, Lifeng Zhao^1^, Hongfeng Gou^3^, Yongmei Xie^1^, Shengyong Yang^1^, Luoting Yu^1^, Li Yang^1,*^, YuquanWei^1,*^.

**Supplementary Figure 1. Nifuroxazide induced apoptosis of CRC cancer cells.** The fluorescence microscopic appearance of Hoechst 33358-stained CT26, HCT116 and HT29 cells after incubated with nifuroxazide for 24 h.


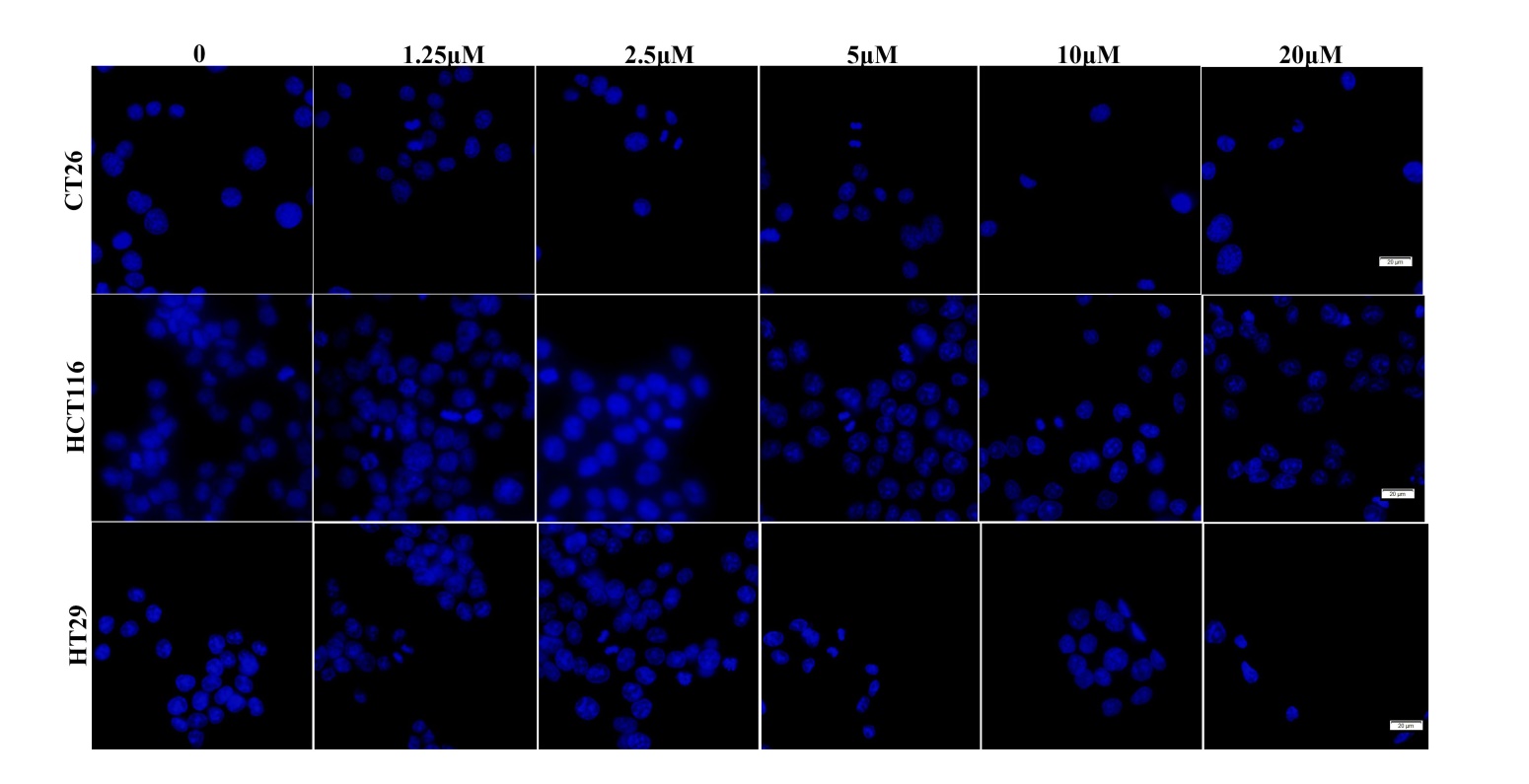

Supplement: Supplementary Figure 1 [file cddis2016452x1.doc]
